# Early-Onset Chronic Drug-Induced Cardiomyopathy in a Pediatric Patient With Ewing Sarcoma

**DOI:** 10.7759/cureus.90840

**Published:** 2025-08-23

**Authors:** Mari Tanaka, Maiko Shimomura, Kosuke Ashihara, Kotaro Urayama, Shuhei Karakawa

**Affiliations:** 1 Pediatrics, Graduate School of Biomedical and Health Sciences, Hiroshima, JPN; 2 Pediatrics, Tsuchiya General Hospital, Hiroshima, JPN

**Keywords:** anthracycline-induced cardiotoxicity, childhood cancer survivors, genetic predisposition, routine surveillance, single nucleotide polymorphisms

## Abstract

Drug-related cardiomyopathy, most notably that caused by anthracyclines, significantly impairs the quality of life in childhood cancer survivors (CCS). In contrast to adults, early-onset chronic cardiomyopathy following chemotherapy is relatively uncommon in CCS, and the underlying pathophysiological mechanisms remain poorly understood. We present a case of a nine-year-old boy who developed severe acute heart failure and subsequent cardioembolic stroke due to early-onset anthracycline-induced cardiotoxicity following treatment for Ewing sarcoma. Whole-exome sequencing revealed two candidate single-nucleotide polymorphisms: HAS3 rs2232228 and RAC2 rs13058338, which may be implicated in the genetic predisposition to anthracycline-related cardiomyopathy. This case highlights the importance of considering early-onset cardiotoxicity in pediatric patients and supports further research into genetic risk-based screening and prevention strategies.

## Introduction

Anthracyclines are the cornerstone agents in pediatric oncology. These drugs are widely used due to their high efficacy in eliminating rapidly dividing cancer cells by intercalating into DNA and inhibiting topoisomerase II [1]. However, cardiotoxicity remains one of their most serious complications in childhood cancer survivors (CCS). Although cardiotoxicity has multiple potential causes, anthracyclines remain one of the primary contributors to complications in CCS [1]. Anthracycline-induced cardiotoxicity (ACT) can lead to long-term cardiac complications, significantly affecting the quality of life and overall health of CCS [1]. ACT is commonly classified into three subtypes as follows: acute (within one week of administration), early-onset chronic (within one year), and late-onset chronic (after one year) [1]. Although early-onset chronic ACT is more common in adults [2], it occurs in only 1.6-2.1% of pediatric patients receiving anthracycline therapy, with late-onset chronic ACT being more typical in children [1]. A cumulative anthracycline dose of ≥250 mg/m² is associated with an increased risk of ACT, and this risk rises in a dose-dependent manner [3]. However, cardiotoxicity has also been reported at lower doses, suggesting that individual susceptibility may be influenced by genetic factors [4,5]. Several genetic variants, including single-nucleotide polymorphisms (SNPs) involved in DNA damage response, oxidative stress, iron metabolism, sarcomere function, and anthracycline metabolism and transport, have been associated with an increased risk of anthracycline-induced cardiomyopathy and validated in multiple studies [4-6]. However, no formal recommendations have been established regarding the integration of genetic information into the clinical decision-making process for cancer patients undergoing anthracycline-based treatment.

This article was previously presented as a meeting abstract at the 66th Annual Meeting of the Japanese Society of Pediatric Hematology/Oncology on December 14, 2024. This case was previously reported by Eto et al. with a focus on the clinical course and management of cardioembolic stroke secondary to ACT [7]. In this report, we present a new perspective by investigating potential genetic predispositions to ACT using whole-exome sequencing.

## Case presentation

**Figure 1:**
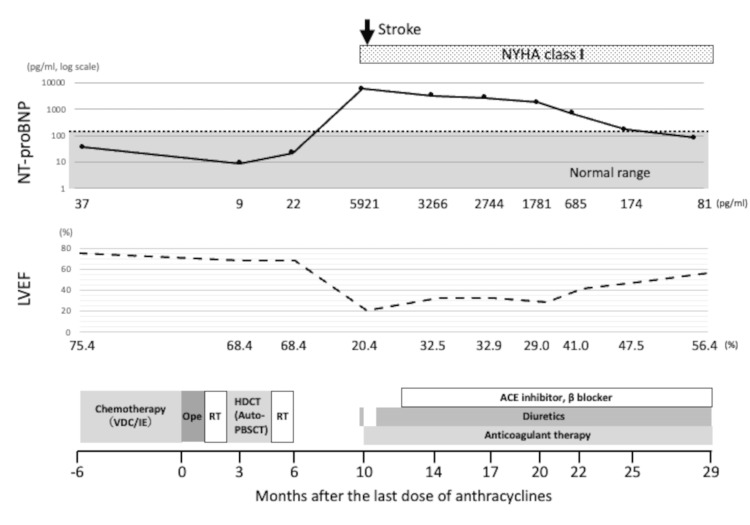
LVEF and NT-proBNP levels measured throughout the patient’s clinical course and during treatment. NYHA: New York Heart Association; Ope: operation; RT: radiation therapy; HDCT: high-dose chemotherapy; PBSCT: peripheral blood stem cell transplantation; ACE: angiotensin-converting enzyme; LVEF: left ventricular ejection fraction; NT-proBNP: N-terminal pro-brain natriuretic peptide NYHA class: I (no symptoms), II (mild symptoms), III (marked limitation), IV (symptoms at rest)

We present a case of a nine-year-old boy diagnosed with Ewing sarcoma originating in the fifth metatarsal bone of the left foot, with metastases to the scapula and bone marrow. He received 11 courses of alternating vincristine, doxorubicin, cyclophosphamide, ifosfamide, and etoposide (VDC/IE) chemotherapy, followed by high-dose chemotherapy with thiotepa and melphalan, and then autologous peripheral blood stem cell transplantation (auto-PBSCT). The cumulative doses included 375 mg/m² for anthracyclines (doxorubicin equivalent) and 66.85 g/m² for alkylating agents (cyclophosphamide equivalent). Subsequently, the patient underwent surgical resection of the primary lesion and radiation therapy to the primary site and right scapula. He achieved complete remission, with cytopenia being the only adverse effect during chemotherapy. Cardiac function remained within normal limits throughout the therapy (Figure 1).

**Figure 2:**
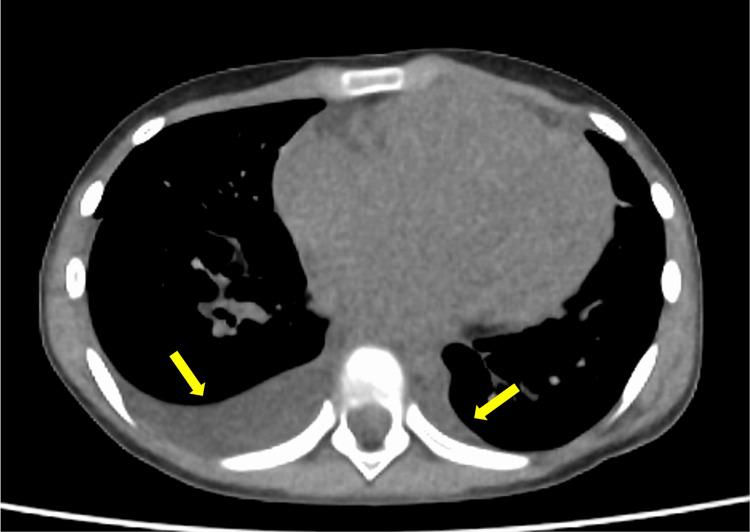
Axial PET-CT image showing pleural effusion (arrows) and cardiomegaly. CT images were acquired with a slice thickness of 4.0 mm. PET imaging was performed 60 minutes after intravenous administration of fluorine-18 fluorodeoxyglucose at a dose of 112 MBq.

**Figure 3:**
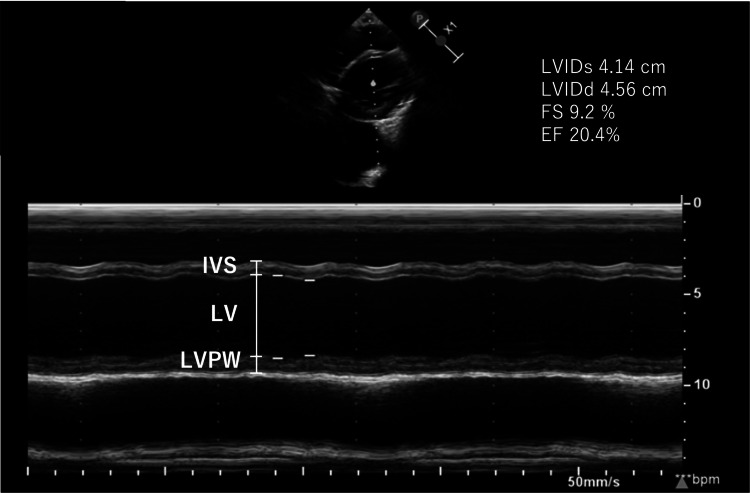
TTE M-mode image at admission. Transthoracic echocardiography was performed using a PHILIPS EPIQ5G ultrasound system (Amsterdam, The Netherlands: Philips) equipped with an S8-3 broadband sector array transducer (3.0-8.0 MHz). TTE: transthoracic echocardiography; LV: left ventricle; IVS: interventricular septum; LVPW: left ventricular posterior wall; LVIDs: left ventricular internal dimension at end-systole; LVIDd: left ventricular internal dimension at end-diastole; FS: fractional shortening; LVEF: left ventricular ejection fraction

However, eight months after the auto-peripheral blood stem cell transplantation (PBSCT) (10 months after the last dose of anthracyclines), routine positron emission tomography-computed tomography revealed cardiomegaly and pleural effusion, suggesting congestive heart failure (Figure 2). Echocardiography revealed a severely reduced left ventricular ejection fraction (LVEF) of 20.4% (Figure 3, Table 1) [8-11].

**Table 1:** Echocardiographic measurements in the present case. *The Z-scores for LVIDs and LVIDd were calculated using the online tool provided by the Boston Children’s Hospital Z-Score Calculator [8], based on normative data described by Sluysmans and Colan [9] and Colan [10]. **Normal ranges of FS and LVEF are based on the study by Tissot et al. [11]. LVIDs: left ventricular internal dimension at end-systole; LVIDd: left ventricular internal dimension at end-diastole; FS: fractional shortening; LVEF: left ventricular ejection fraction; NA: not applicable

Variables	Measurement value	Reference percentile/Z-score	Normal range
LVIDs (cm)	4.14	>98th percentile (+6.88 SD)*	NA
LVIDd (cm)	4.56	>98th percentile (+2.44 SD)*	NA
FS (%)	9.2	NA	28-46**
LVEF (%)	20.4	NA	55-75**

At presentation, the patient’s vital signs were stable, and he did not complain of any exertional symptoms, such as palpitations or dyspnea, mostly because of the limited physical activity secondary to surgery-related gait impairment. Laboratory findings revealed a markedly elevated N-terminal pro-brain natriuretic peptide (NT-proBNP) level of 5921 pg/mL (normal: <160 pg/mL for individuals aged 6-18 years [12]), with otherwise unremarkable findings (Figure 1). Accordingly, the patient was classified as New York Heart Association (NYHA) class I. Viral myocarditis was not considered due to the absence of recent viral illness. There was no family history of cardiomyopathy or sudden cardiac death. The patient had an adequate nutritional status. Moreover, endocrine evaluations, including thyroid function, were within normal limits.

**Figure 4:**
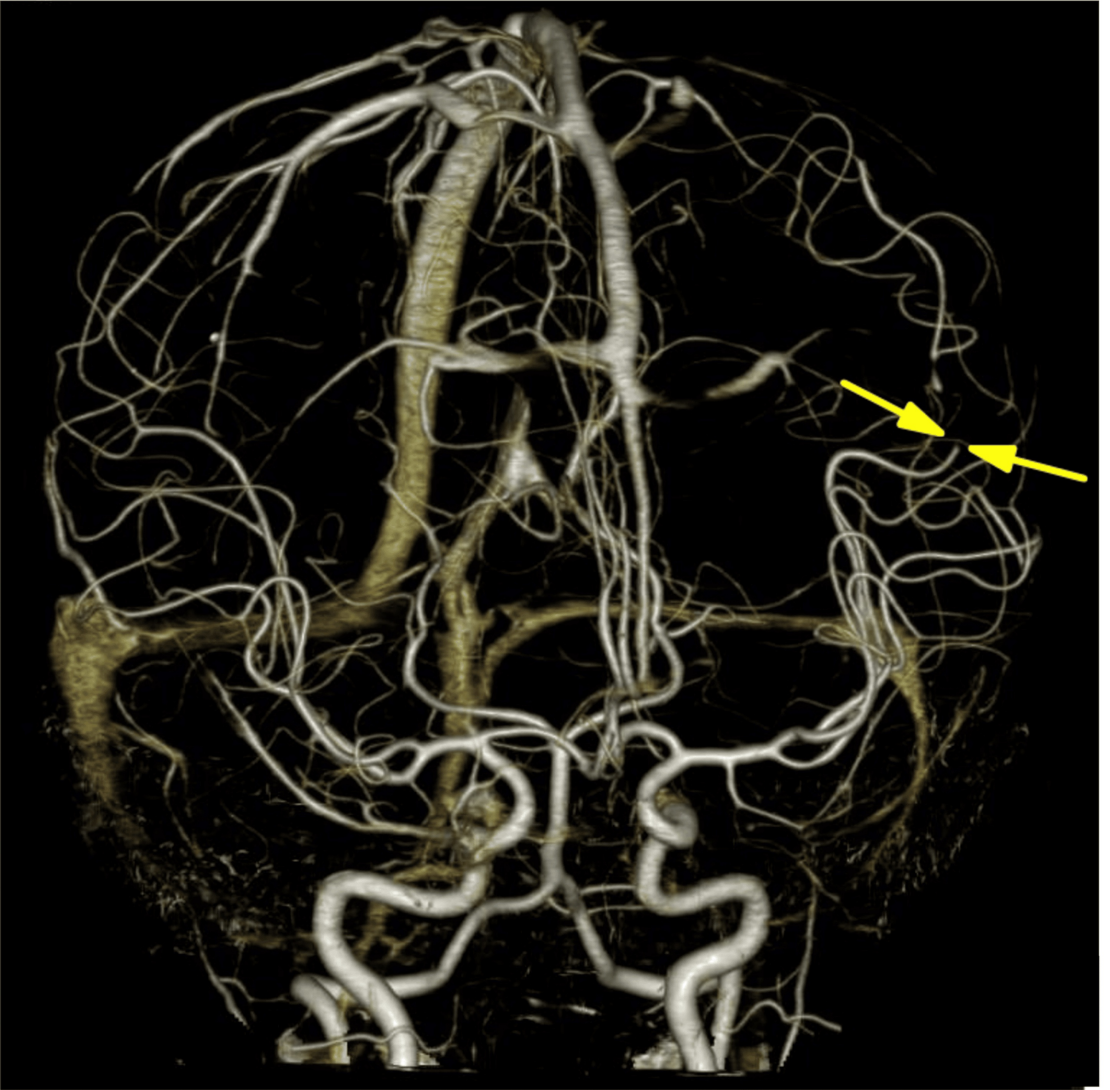
CTA showing obstruction of the left middle cerebral artery on day four of hospitalization (arrows). CTA: computed tomography angiography

**Figure 5:**
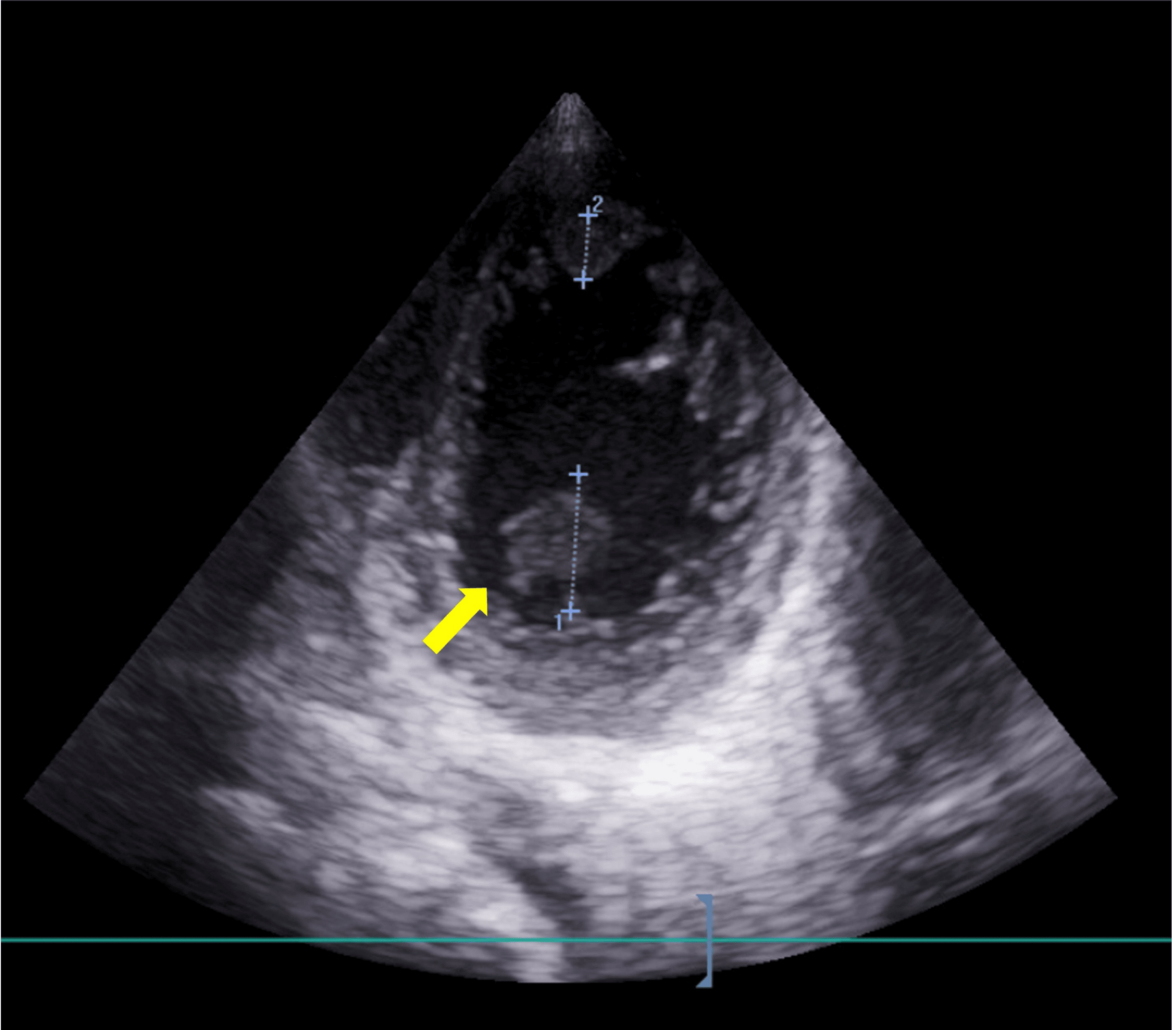
Transthoracic echocardiography image. Parasternal short-axis transthoracic echocardiography (TTE) image showing a mobile left ventricular thrombus (LVT) on day four of hospitalization (arrow) [7].

**Figure 6:**
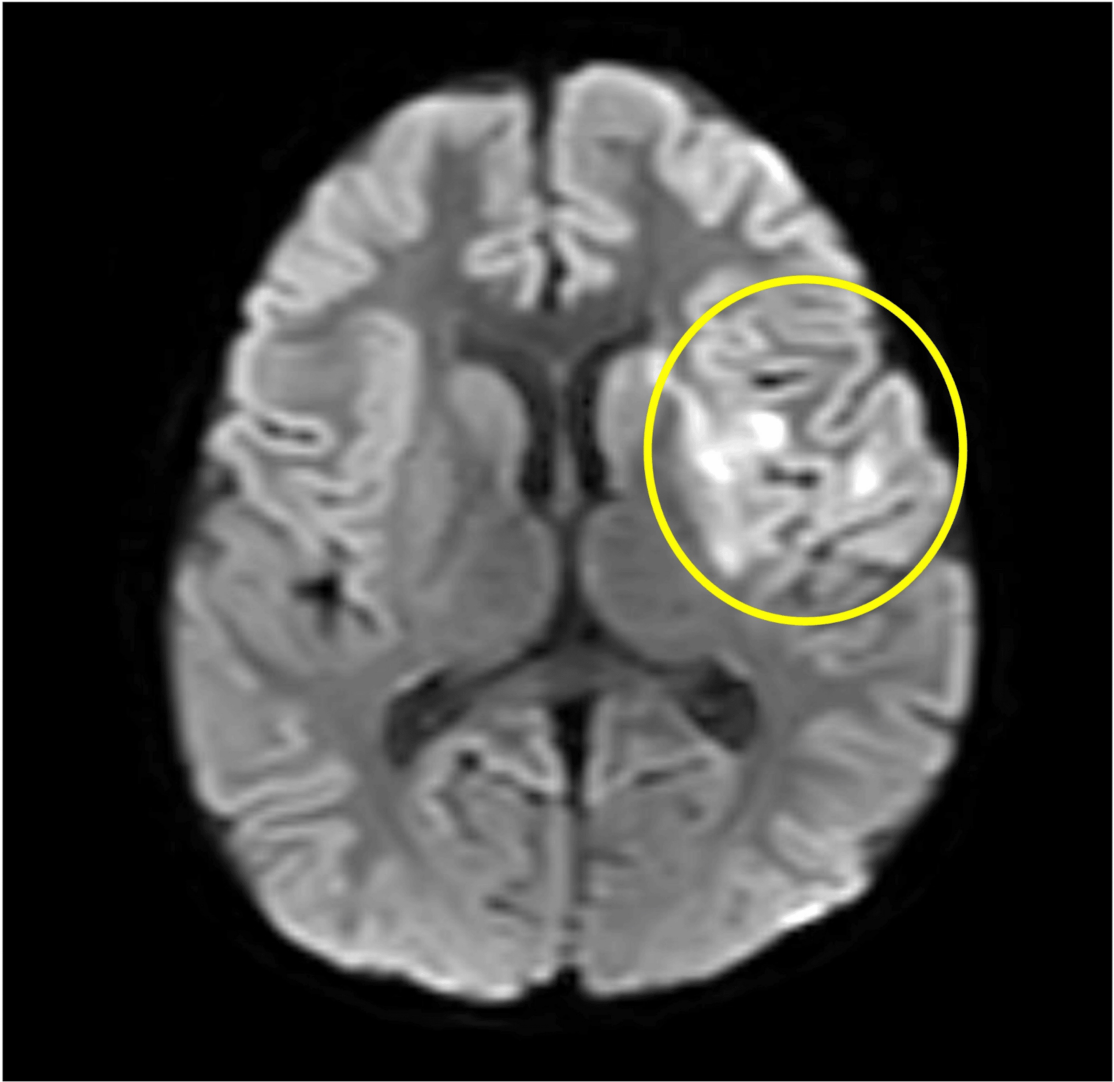
Diffusion-weighted magnetic resonance imaging (3.0T, b=1000 s/mm², slice thickness 5 mm) performed 24 hours after onset of cerebral infarction. The image shows high-signal-intensity lesions in the middle cerebral artery territory (circled area).

The absence of dyspnea or chest pain reduced the likelihood of ischemic heart disease. Based on the patient’s treatment history, he was diagnosed with acute heart failure due to early-onset chronic anthracycline-induced cardiotoxicity (ACT), and diuretic therapy was initiated. Four days later, he developed complete right hemiparesis, leftward conjugate gaze deviation, and aphasia. Computed tomography angiography revealed occlusion of the left middle cerebral artery (Figure 4), and echocardiography confirmed a thrombus in the left ventricle, which had not been detected at presentation (Figure 5) [7]. This patient had no evidence of thrombophilia, congenital heart defects, vascular abnormalities, hypertension, or dyslipidemia that could predispose to atherosclerotic thrombi formation. A diagnosis of cardioembolic stroke was made. Twenty-four hours after the onset of stroke symptoms, diffusion-weighted imaging revealed a high-intensity signal in the left middle cerebral artery region (Figure 6). The patient received prompt thrombolytic therapy and was discharged without any neurological sequelae. These clinical events and treatments have been previously described in detail [7].

Nineteen months after the onset of heart failure, the patient’s LVEF gradually improved to 56.4%; however, he remained on heart failure therapy and had limited physical activity (Figure 1). Given the early onset and the severe clinical course of ACT, we suspected a genetic predisposition. Since there is no commercially available targeted genetic testing panel specific to ACT and only limited overlap between ACT and other inherited forms of cardiomyopathy, we selected whole-exome sequencing (WES) to allow for an unbiased and comprehensive assessment of potential genetic predisposition in this case. Accordingly, informed consent for genetic analysis was obtained from the patient’s family (IRB #E2107-9201). WES identified two candidate single-nucleotide polymorphisms (SNPs): a heterozygous variant in* *HAS3* *(NM_001199280.2), c.279A>G (p. Ala93=), rs2232228 [13], and a heterozygous variant in* *RAC2 (NM_002872.5), c.108-3812A>T, rs13058338 [14].

## Discussion

In pediatric patients, late-onset ACT is frequently observed, and most pediatric cardiotoxicity surveillance protocols, including those outlined in long-term follow-up guidelines for CCS, are designed with a focus on late-onset manifestations [3].

ACT often presents initially as asymptomatic left ventricular systolic or diastolic dysfunction (ALVD), which may progress subclinically. Symptomatic heart failure has been documented in approximately 10.6% of the CCS population. The prevalence of ALVD is not well-defined, with some studies suggesting rates as high as 28% [15]. Although pharmacological intervention is recommended for adult patients with an LVEF <40%, there is no consensus regarding the management of asymptomatic individuals with higher LVEF values [16]. Furthermore, data on early-onset ACT in pediatric populations are scarce, underscoring the need for more robust evidence in this area [3]. Although our patient was considered at high risk for late-onset ACT due to a cumulative dose, the development of early-onset ACT could not have been anticipated, raising suspicion of an underlying genetic predisposition. WES revealed two single-nucleotide polymorphisms (SNPs) that were previously implicated in ACT susceptibility as follows:* *HAS3 rs2232228 and* *RAC2 rs13058338 [13,14]. The GA genotype at HAS3 rs2232228 (first SNP detected in our patient) was associated with an increased risk of cardiomyopathy in a CCS cohort following high-dose anthracycline exposure (>250 mg/m²) compared with the GG genotype (odds ratio: 3.6; p=0.07) [13].HAS3* *encodes hyaluronic acid, a key component of the extracellular matrix that is involved in post-injury cardiac remodeling [13]. Additionally, the TA genotype at RAC2 rs13058338 (second SNP detected in our patient) was also found to be associated with an elevated risk of heart failure among hematopoietic cell transplantation survivors (odds ratio: 2.8; p<0.01) [14]. RAC2 encodes a GTPase essential for NAD(P)H oxidase activity and reactive oxygen species generation, which are involved in anthracycline cardiotoxicity [14]. The global allele frequency of this variant is approximately 0.20, with a notably lower frequency in the Japanese population (0.09), as reported in the gnomAD (https://gnomad.broadinstitute.org/) and the ToMMo 54KJPN databases (https://jmorp.megabank.tohoku.ac.jp). Although specific SNPs have been linked to ACT, their exact contributions and ethnic variability remain poorly understood. Integrating genetic and clinical risk factors in a multivariable prediction model may enhance the clinical utility of genetic screening. Although several promising genetic risk prediction models combining clinical variables, such as age at treatment initiation, cumulative anthracycline dose, sex, chest radiotherapy, and ethnicity, have been developed, a robust and clinically applicable model incorporating time-to-event data has yet to be established [17-19]. Chaix et al. reported that the integration of WES data with a random forest model significantly improved the accuracy of predicting ACT in CCS [20]. However, further validation in global multicenter cohorts is necessary to develop reliable predictive models that can guide individualized risk assessment and early intervention strategies. Therefore, establishing large-scale registry studies incorporating genetic testing, along with prospective screening protocols based on such data, is warranted to optimize risk-adapted strategies and long-term follow-up models.

In addition to these advances, the routine implementation of WES in CCS presents both potential benefits and challenges. From a cost-effectiveness perspective, although WES can provide comprehensive genetic insights that may inform personalized surveillance and intervention strategies, the high upfront costs and limited accessibility remain significant barriers, particularly in resource-constrained settings. Moreover, ethically, using WES raises important considerations, including informed consent, management of incidental findings, and the psychological impact of genetic information on patients and their families. Therefore, careful deliberation is required to balance the clinical utility of routine WES and its financial burden and ethical implications.

## Conclusions

We encountered a pediatric case of early-onset chronic ACT complicated by cardioembolic stroke. This case illustrates a clinically significant but underrecognized form of early-onset chronic ACT that can occur prior to routine surveillance. Although this report describes a single case, the presence of genetic variants that potentially predispose patients to ACT highlights the urgent need for research on individualized risk assessment and early intervention strategies in pediatric oncology. The development of evidence-based screening protocols that incorporate genetic risk factors may ultimately help reduce the morbidity associated with anthracycline cardiotoxicity in children.
